# Non-invasive Ventilation in Children With Neuromuscular Disease

**DOI:** 10.3389/fped.2020.00482

**Published:** 2020-11-16

**Authors:** Brigitte Fauroux, Sonia Khirani, Lucie Griffon, Theo Teng, Agathe Lanzeray, Alessandro Amaddeo

**Affiliations:** ^1^Pediatric Non-invasive Ventilation and Sleep Unit, AP-HP, Hôpital Necker-Enfants Malades, Paris, France; ^2^Université de Paris, VIFASOM, Paris, France; ^3^ASV Sante, Gennevilliers, France

**Keywords:** non-invasive ventilation, child, neuromuscular disease, nocturnal hypoventilation, sleep, sleep-disordered breathing, home treatment

## Abstract

The respiratory muscles are rarely spared in children with neuromuscular diseases (NMD) which puts them at risk of alveolar hypoventilation. The role of non-invasive ventilation (NIV) is then to assist or “replace” the weakened respiratory muscles in order to correct alveolar hypoventilation by maintaining a sufficient tidal volume and minute ventilation. As breathing is physiologically less efficient during sleep, NIV will be initially used at night but, with the progression of respiratory muscle weakness, NIV can be extended during daytime, preferentially by means of a mouthpiece in order to allow speech and eating. Although children with NMD represent the largest group of children requiring long term NIV, there is a lack of validated criteria to start NIV. There is an agreement to start long term NIV in case of isolated nocturnal hypoventilation, before the appearance of daytime hypercapnia, and/or in case of acute respiratory failure requiring any type of ventilatory support. NIV is associated with a correction in night- and daytime gas exchange, an increase in sleep efficiency and an increase in survival. NIV and/or intermittent positive pressure breathing (IPPB) have been shown to prevent thoracic deformities and consequent thoracic and lung hypoplasia in young children with NMD. NIV should be performed with a life support ventilator appropriate for the child's weight, with adequate alarms, and an integrated (±additional) battery. Humidification is recommended to improve respiratory comfort and prevent drying of bronchial secretions. A nasal interface (or nasal canula) is the preferred interface, a nasobuccal interface can be used with caution in case of mouth breathing. The efficacy of NIV should be assessed on the correction of alveolar ventilation. Patient ventilator synchrony and the absence of leaks can be assessed on a sleep study with NIV or on the analysis of the ventilator's in-built software. The ventilator settings and the interface should be adapted to the child's growth and progression of respiratory muscle weakness. NIV should be associated with an efficient clearance of bronchial secretions by a specific program on the ventilator, IPPB, or mechanical insufflation-exsufflation. Finally, these children should be managed by an expert pediatric multi-disciplinary team.

## Introduction

Long term non-invasive ventilation (NIV) involves the delivery of ventilatory assistance through a non-invasive interface, as opposed to invasive ventilation via a tracheostomy. Children with neuromuscular disease (NMD) represent the largest group of children requiring long term NIV (1). Indeed, the respiratory muscles are rarely spared in children with NMD which puts them at risk of alveolar hypoventilation, especially during sleep. The role of NIV is then to assist or “replace” the weakened respiratory muscles in order to correct alveolar hypoventilation by maintaining a sufficient tidal volume and minute ventilation (2). Despite the large use of NIV in children with NMD, there is a lack of validated criteria to start NIV and follow up relies mainly on experience and practice (1, 2). NIV is associated with a correction in night- and daytime gas exchange, an increase in sleep efficiency and an increase in survival but data on quality of life are scarce (1, 3–6). In children, the technological aspects of NIV are crucial with the need of regular adaptations according to the patient's age and disease progression. This review aims at giving an update on the different aspects on NIV in these severely disabled children and underlines the importance of a management and follow up by an expert pediatric multidisciplinary team.

## Why May Some Children With Neuromuscular Disease Need NIV?

**Figure 1:**
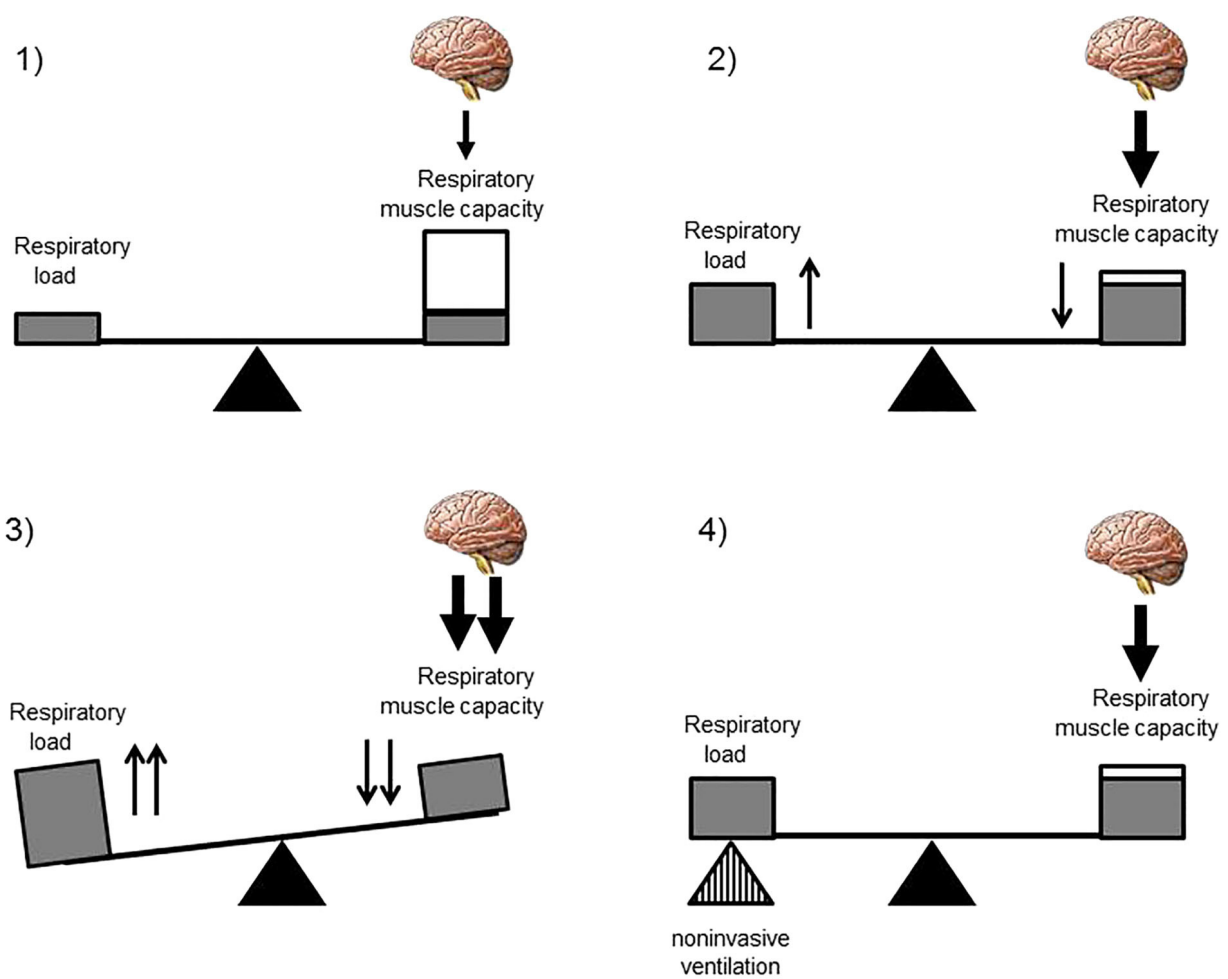
Disequilibrium of the respiratory balance in children with neuromuscular disease. (1) The normal respiratory balance comprises the load imposed on the respiratory system, the capacity of the respiratory muscles, and the central drive. In a healthy subject, the respiratory load is low, the capacity of the respiratory muscles is normal, and the brain appropriately commands the respiratory muscles. Only a small proportion of the total respiratory muscle capacity is necessary for the breathing process. (2) In children with neuromuscular diseases, the respiratory load is increased and the capacity of the respiratory muscles is decreased. The brain drives the weakened respiratory muscles which need to use a large proportion of their total capacity for the breathing process. (3) Excessive weakness of the respiratory muscles and/or an increase in the respiratory load causes disequilibrium in the respiratory balance which translates into alveolar hypoventilation if the imbalance exceeds a certain threshold. (4) Non-invasive ventilation may correct this disequilibrium by assisting or “replacing” the weakened respiratory muscles.

In healthy subjects, the respiratory load, i.e., the effort the subject has to perform to generate a breath, is low, the capacity of the respiratory muscles is normal, and the central drive appropriately commands the respiratory muscles (Figure 1). In disorders characterized by a weakness of the respiratory muscles as observed in NMD, the central drive increases its demands of the respiratory muscles. However, when the respiratory muscles are not able to cope with the respiratory load, hypoventilation, defined by hypercapnia and hypoxemia, occurs. NMD that involve the motor neuron, the peripheral nerve, the neuromuscular junction, or the muscle may cause excessive respiratory muscle weakness. Kyphoscoliosis, which is common in children with NMD, may increase the respiratory load and cause a mechanical disadvantage of the respiratory muscles, precipitating alveolar hypoventilation.

The most common NMD requiring NIV during childhood are Duchenne muscular dystrophy (DMD) and spinal muscular atrophy (SMA). DMD is a progressive disorder, and ventilatory failure is inevitable in the course of the disease, although the time course of progression to it varies between individuals. Alveolar hypoventilation is also common in children with SMA type I or II. Alveolar hypoventilation is less frequent in other muscular dystrophies, such as Becker, limb-girdle, and facioscapulohumeral dystrophies. Congenital myopathies are often less progressive (7). However, some congenital myopathies such as collagen 6 (COL6) myopathies or selenopathies are characterized by a predominant weakness of the diaphragm, exposing these children to sleep-disordered breathing, although their peripheral muscle strength remains relatively preserved (8, 9). The importance of respiratory failure associated with spinal cord injury depends on the level of the injury. High spinal cord injury, above C3, causes diaphragm paralysis. In patients with lower cervical cord injury, expiratory muscle function is compromised, impairing cough and the clearance of bronchial secretions. As a result, the retention of secretions leading to atelectasis and bronchopneumonia frequently occurs. It has to be noted that the respiratory status of children with NMD may deteriorate with growth because the weakened muscles may be unable to cope with an increasing body mass and metabolic demand.

**Figure 2:**
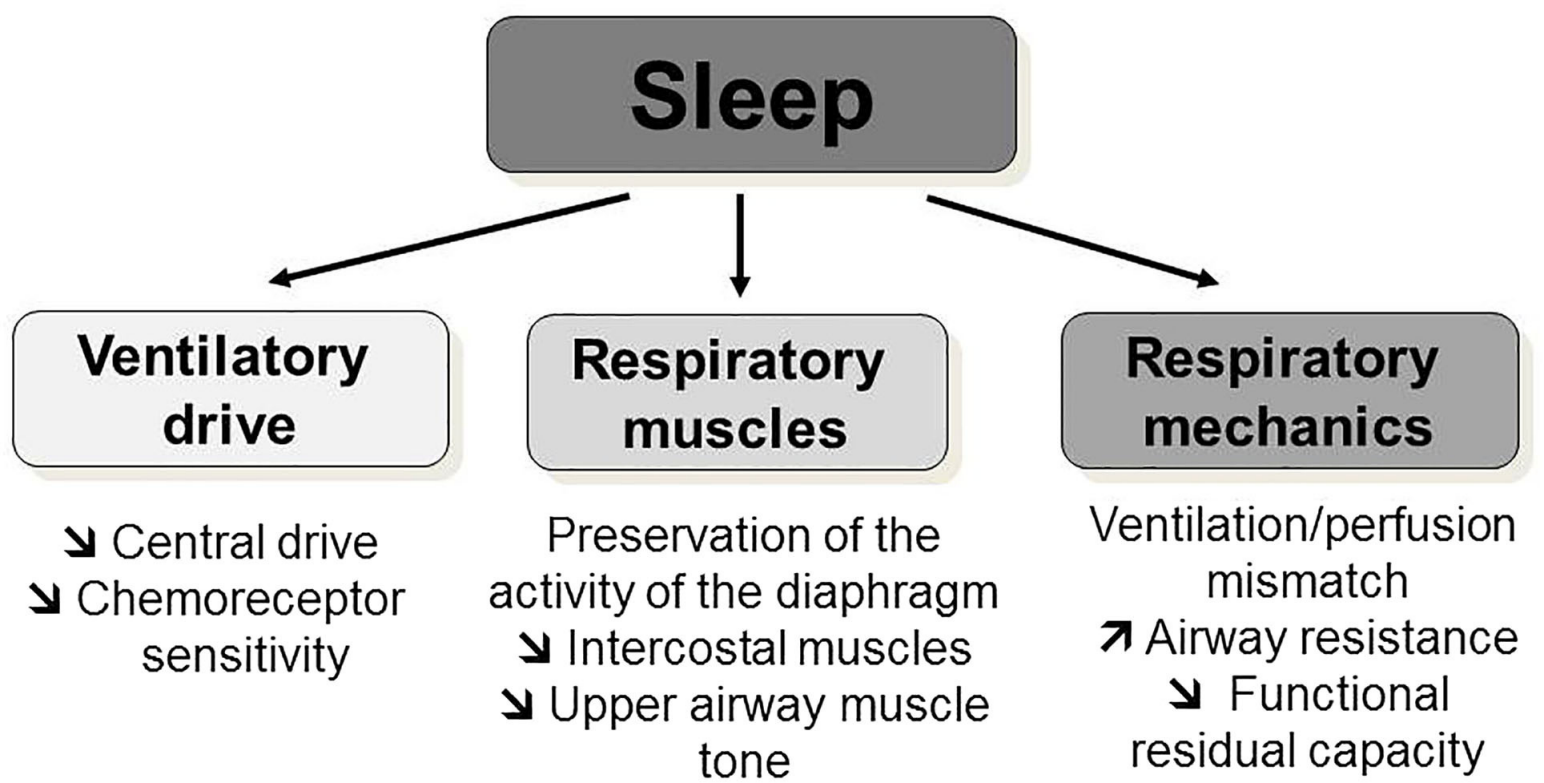
Physiological respiratory changes during sleep. Sleep is associated with physiological changes: a decrease in central drive and chemoreceptor sensitivity; a decrease of the intercostal and the upper airway muscles activity and tone with a preservation of the activity of the diaphragm; and an increase in ventilation–perfusion mismatch, in airflow resistance, and a fall in functional residual capacity.

**Figure 3:**
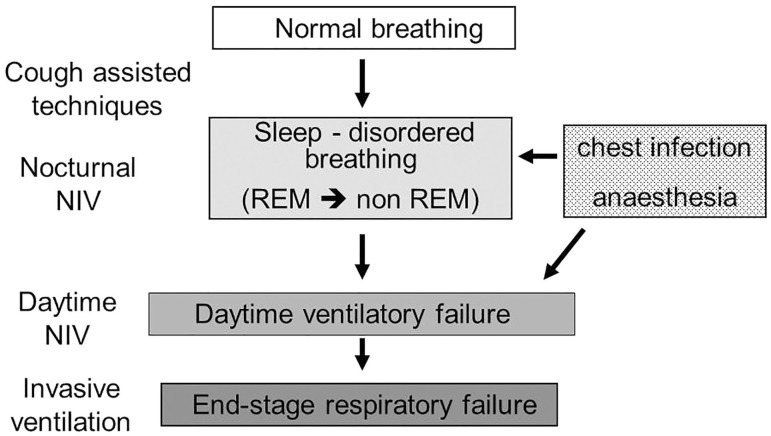
Progression of sleep-disordered breathing toward respiratory failure progressive neuromuscular disorders. Nocturnal hypoventilation during rapid-eye movement sleep is the first breathing abnormality that a patient with neuromuscular diseases may develop during the progression of the weakness of their respiratory muscles. Cough assisted-techniques should be implemented at an early stage, before the onset of nocturnal hypoventilation. Even at an early stage, acute respiratory failure can be precipitated by a respiratory infection or an anesthetic procedure, underlying the importance of preventative measures. With the progression of respiratory muscle weakness, the patient will develop continuous nocturnal and daytime hypoventilation. Daytime ventilation may postpone the discussion of a tracheotomy, which will need to be discussed at the time of end-stage respiratory failure. REM, rapid-eye movement; NIV, non-invasive ventilation.

NIV is a non-invasive ventilator assistance that assists the breathing of the patient by delivering a positive pressure during each inspiration in order to maintain a sufficient tidal volume and minute ventilation. In NMD, the role of NIV is to assist or “replace” the weakened respiratory muscles in order to restore a normal breathing and correct alveolar hypoventilation. In progressive NMD, NIV is initiated and preferentially used during sleep. Indeed, sleep is associated with changes in respiratory mechanics with an increase in ventilation–perfusion mismatch and in airflow resistance and a fall in functional residual capacity (Figure 2). Although the activity of the diaphragm is preserved, that of the intercostal and the upper airway muscles significantly decreases. Finally, central drive and chemoreceptor sensitivity are less efficient during sleep than during wakefulness. All these physiological changes explain why the arterial pressure in carbon dioxide (PaCO_2_) may rise of up to 3 mmHg (0.4 kPa) in healthy subjects. This decrease in alveolar ventilation predominates during rapid eye movement sleep and explains the greater vulnerability of patients with NMD during this sleep stage. The majority of children with NMD need NIV only during sleep (10, 11) but some children with progressive NIV may develop daytime respiratory failure, requiring additional ventilator support during daytime (Figure 3) (12).

## When Should NIV Be Started?

There are no validated criteria to start long term NIV in children (Figure 3). In clinical practice, NIV may be initiated in an acute setting, after invasive or NIV weaning failure in the pediatric intensive care unit, in a subacute or chronic setting, on abnormal nocturnal gas exchange alone or on the association of abnormal gas exchange and respiratory events on a polysomnography (1, 13, 14).

In clinical practice, consensus conferences agree on the value of daytime hypercapnia and recurrent acute respiratory exacerbations to initiate NIV because these criteria are the signature of established ventilatory failure (15). But these classical criteria are preceded by a variable period of nocturnal hypoventilation during which treatable symptoms, such as frequent arousals, poor sleep quality, severe orthopnoea, daytime fatigue and alterations in cognitive function, may deteriorate the daily life of the patient (6, 16, 17).

The main challenges or difficulties for NIV initiation in children are thus to define (1) the timing and type of investigation, such as a polysomnography, a polygraphy, or an overnight gas exchange recording, that should be performed for NIV initiation, and (2) the values or thresholds of the parameters that should be retained for NIV initiation, such as the oxygen (O_2_) and/or CO_2_ level, and/or apnea-hypopnea index (AHI), with the assumption that their correction will be associated with a benefit of NIV (14). These difficulties are due to the lack of markers of end-organ morbidity associated with sleep-disordered breathing and chronic respiratory failure in children with NMD.

NIV may be justified without a sleep study when the child presents episodes of acute respiratory failure triggered by a respiratory infection or an anesthetic procedure, as these events are markers of an insufficient respiratory reserve (14, 15). Concerning the timing of a sleep study, there is a lack of validated recommendations. This may be in part explained by the heterogeneity of pediatric NMD and the variability of respiratory involvement within a specific disease such as SMA or COL6 myopathies (18, 19). Symptoms of sleep-disordered breathing may be silent or difficult to establish in children because of reliance on parents and second-hand caregivers, who may have difficulties assessing the child's disease and sleep. Children with a progressive NMD tend to underestimate symptoms such as fatigue before using NIV because onset is generally insidious. Most importantly, symptoms suggestive of sleep-disordered breathing did not differ between children with a NMD with or without documented nocturnal hypoventilation (17). Symptoms cannot thus be used as predictors or markers of nocturnal hypoventilation. Lung function parameters are also poor indicators of nocturnal hypoventilation. In patients with NMD, vital capacity (VC) and inspiratory VC have been shown to have some correlation with daytime and nocturnal gas exchange (20, 21). Daytime predictors of nocturnal hypoventilation have mainly been identified for patients with DMD who represent a relative homogeneous group of patients. As such, the forced expiratory volume in 1 s (FEV1), daytime PaO_2_ and PaCO_2_, base excess and the rapid shallow breathing index were all significantly correlated to nocturnal hypoventilation in patients with DMD (21). Another study in children with various NMD did not identify a sensitive and specific daytime lung function or respiratory muscle test that was associated with, or predictive of, nocturnal hypoxemia or hypercapnia (22). In clinical practice, it seems important to take into account the type of NMD as nocturnal hypoventilation occurs preferentially in disorders characterized by a prominent diaphragmatic weakness (23). As such children with a COL6 myopathy or a selenopathy should be screened systematically for sleep disordered breathing (8, 9). Prioritized screening is also recommended for infants or young children with congenital myopathies or rapidly progressive NMD (24).

In children with NMD, the documentation of nocturnal hypoventilation by means of a polysomnography is recommended but not mandatory prior to starting NIV because “isolated” abnormal nocturnal gas exchange may be sufficient (25). Indeed, a prospective evaluation of 10 children and adults with a NMD or a thoracic deformity and isolated nocturnal hypercapnia without daytime hypercapnia showed that 9 patients progressed to overt daytime respiratory failure within a period of 2 years (25). However, in the absence of a validated definition of alveolar hypoventilation, different definitions are used in the literature, leading to a different prevalence and severity of alveolar hypoventilation (26). Moreover, the scoring of respiratory events by the American Academy of Sleep Medicine (AASM) is not adapted for patients with NMD (27). Indeed, apneic events are rare in patients with NMD. Progressive simultaneous decrease in airflow and thoracic and abdominal movements, suggestive of global inspiratory muscle weakness, are more common but not scored as respiratory events unless they are accompanied by a (micro)-arousal or a desaturation (28). Paradoxical breathing with opposition phase on the thoracic and abdominal belts may be the consequence of diaphragmatic dysfunction or weakness of the intercostal muscles and should not be falsely scored as an “obstructive event” (28–30). In clinical practice, periods of “reduced ventilation” or paradoxical breathing, more than obstructive and/or central apnea-hypopneas, especially during rapid-eye movement sleep, associated with a pulse oximetry (SpO_2_) <90% and/or a transcutaneous CO_2_ (PtcCO_2_) value > 50 mmHg are indicative of an insufficient respiratory muscle performance and should discuss the initiation of long term NIV in children with NMD.

In conclusion, screening with at least an overnight gas exchange recording to detect nocturnal hypoxemia and/or hypercapnia, and if possible with a more complete sleep study, should be a priority in all children with any NMD that may be associated with nocturnal hypoventilation. Symptoms of sleep-disordered breathing are insufficiently sensitive and specific and tend to appear late in the course of NMD (17). For the future, the determination of the efficacy of NIV according to the different clinical scenarios and the underlying disease seems important. Larger prospective studies, in homogeneous group of patients with NMD, are also warranted to confirm the benefit of the initiation of NIV at the stage of “isolated” nocturnal hypoventilation.

A pre-operative training with NIV and a cough assisted technique before a planned surgical intervention, such as a spinal fusion, has been shown be very effective to improve the post-operative respiratory outcome and reduce the incidence of respiratory complications in selected children with NMD and cerebral palsy (31).

## What Are the Benefits of NIV?

The large use of NIV in children with NMD contrasts with the limited number of studies that have evaluated the benefits of NIV in children. Studies involving a small number of patients have shown that NIV is associated with a correction of nocturnal and daytime gas exchange, improved sleep quality, and reduced symptoms associated with sleep-disordered breathing (4–6). NIV has also been associated with an increase in survival in patients with DMD in case series and a nationwide study in Denmark. Indeed, an analysis of the national DMD register in Denmark showed that mortality significantly fell between 1977 and 2001 due to the large increase in ventilator users (32). NIV, associated with nutritional support and cough-assisted techniques, has also been shown to increase survival in infants with SMA type I (33, 34). NIV was also associated with an improvement in the quality of life in infants with SMA and boys with DMD (3, 16, 35, 36).

NIV, associated with mechanical insufflation-exsufflation has been shown to prevent thoracic deformities and consequent thoracic and lung hypoplasia in young children with NMD (37). Intermittent positive pressure breathing (IPPB), which consists in the delivery of intermittent high inspiratory pressures, usually on a daily basis, has been shown to increase ventilation in patients with NMD (38). Both NIV and IPPB technique are also efficient to prevent atelectasis and the risk of pneumonia in children with NMD (39). In clinical practice, NIV is associated with improved feeding, weight gain and growth, which may be related to a decrease of the work of breathing and consequent caloric burn and improved eating and swallowing (6). Neurocognitive dysfunction and behavioral disturbances are the most common and severe consequences of obstructive sleep apnea (OSA) in children (40) but this aspect has been less studied in children with NMD. Patient-reported benefits of mouthpiece ventilation associated a reduction in dyspnea and fatigue as well as an improvement in speech and eating in a study of 30 patients (41).

## Which NIV Equipment and Settings Are Recommended?

NIV equipment comprises the interface, the circuit and the device. During NIV, a higher level of positive pressure is delivered during inspiration, by means of a volume- or pressure-targeted mode. The first ventilators used for patients with NMD delivered a volume-targeted ventilation, characterized by the generation of a fixed inspired volume during a given time span. The advantage of this mode is the strict delivery of the preset volume. Its main disadvantage is that this mode is not able to adjust to the variable requirements of the patient, such as physiological changes in central drive, lung compliance and airway resistance during sleep. Importantly, compensation for unintentional leaks is not always possible with this mode, which exposes the patient to the risk of an insufficient effective inspired volume in the presence of unintentional leaks (42). Consequently, nowadays, the ventilator mode that is the most used is a pressure-targeted mode, eventually with a target inspired volume in order to overcome the limitations of volume-targeted ventilation with a single-limb circuit. During this mode, the ventilator measures or estimates each consecutive expired volume and automatically adjusts inspiratory pressure within a predetermined range to ensure a stable target volume (43).

The settings of NIV have to be individually-adjusted to the patient. In children with NMD, the aim is to deliver a physiological tidal volume of ~8–10 ml/kg. This can be achieved with low inspiratory pressures in young infants having compliant lungs and chest wall, but higher inspiratory pressures may be necessary in older children, those with scoliosis and/or obesity. Expiratory pressures should be set at the lowest values, as patients with NMD usually do not have airway obstruction. A back up rate close to the physiological breathing rate during sleep is recommended in order to limit the inspiratory triggering of the ventilator by the patient, which can be difficult for a child with weakened respiratory muscles. It has to be noted that continuous positive airway pressure (CPAP) is clearly NOT the treatment of sleep-disordered breathing in patients with NMD. Humidification of the circuit is recommended to improve respiratory comfort and prevent drying of bronchial secretions.

Interfaces for NIV may cover the nose (nasal mask), the nose and the mouth (nasobuccal mask), the face (total face mask) and for daytime use, the mouth only (mouthpiece) (44, 45). Nasal pillows (or prongs or cannulas) are minimal contact interfaces which are available for school-aged children and are very well-tolerated (44). Interfaces can be vented, meaning that they incorporate intentional leaks to be used with a single limb circuit and a preset minimal positive expiratory pressure, and non-vented masks, which can be used with a double limb circuit or a single limb circuit with an expiratory valve, and with or without a positive expiratory pressure. A minimal level of expiratory pressure is mandatory for vented masks, in order to allow the clearing of CO_2_ during expiration (46, 47). The choice of the interface is determined by the patient's age, weight, facial and skull anatomy for the fitting of the headgear, nasal permeability and the eventual presence of mouth breathing, ventilator (requiring a vented or non-vented interface), comfort and tolerance of the interface, and the patient's ability to remove the interface by him(her)self. In children requiring NIV during daytime, NIV may be performed with the interface used during sleep but also a mouthpiece (12, 41, 48). The advantages, disadvantages and side effects of the different interfaces are listed in Table 1.

**Table 1:** Advantages, disadvantages and side effects of interfaces for children.

**Interface**	**Advantages**	**Disadvantages**	**Side effects**
Nasal mask	Small internal volume Large choice	Not usable in case of mouth leaks	Pressure sores, eye irritation if leaks, maxillary retrusion
Nasobuccal mask	Prevents mouth leaks	Large volume, risk of inhalation of gastric content in case of oesogastric reflux, no model available for infants	Pressure sores, eye irritation if leaks, facial deformity
Total face mask	Prevents mouth leaks	Large volume, risk of inhalation of gastric content in case of oesogastric reflux	Pressure sores, facial deformity
Nasal prongs	Small, light No pressure sores	Not usable in case of mouth leaks, no model available for infants	Nasal irritation
Mouthpiece	Small and light, no pressure sores, to be used on demand	Not usable during sleep	None

NIV should be performed with a life support ventilator appropriate for the child's weight, with adequate alarms, and an integrated (± additional) battery. Humidification is recommended to improve respiratory comfort and prevent drying of bronchial secretions. NIV has to be associated with an efficient clearance of bronchial secretions by either, a specific program on the ventilator, IPPB, or mechanical insufflation-exsufflation. The patient and his caregivers should be trained to these clearance techniques in order to prevent or limit bronchial encumbrance and respiratory exacerbations.

## How Should Children With NIV Be Followed?

NIV is a technically challenging treatment which aim is to be performed at home. NIV is usually initiated in the hospital during a short hospitalization of 2 or 3 nights in order to progressively acclimatize the patient to his (her) NIV treatment. As NIV will be administered during sleep, an overnight monitoring of sleep with the optimal setting(s), at least with an assessment of overnight gas exchange, and ideally with polygraphy or polysomnography, is recommended before discharge. This sleep study can be postponed until the patient is well-adapted to NIV and is able to sleep at least 6 h with his device. Training of the caregivers and of the patient is essential. The caregivers must be familiar with the putting on and taking off of the NIV interface and device and should have an appropriate training on the different problems that may occur at home (49). Careful checking of the caregiver competencies before discharge is mandatory. In order to cope with the shortage of hospital beds and the demands of the families, it is possible to started NIV in an out-setting in selected patients with an efficacy and compliance comparable to an in-hospital initiation (13, 50, 51). Home visits by trained nurses and/or technicians are possible in some countries and constitutes a major factor of the success of a home NIV program. Indeed, compliance with NIV is a major issue as treatment efficiency is related to the length of nocturnal use (52). Most studies have reported relatively low compliance rates with mean night uses between 3 and 5 h (52–54). However, objective compliance levels close to the recommended sleep duration in children can be achieved by expert centers having a specific NIV therapeutic education program (55). The caregivers should be able to contact the home care provider for any technical assistance and the hospital team for any medical issue at any time (49).

There are no validated guidelines for the monitoring or long term follow up of children with NIV. The timing of the follow up visits depends on the age and the medical condition of the child. Our practice consists in a sleep study 1 month after the initiation, and then every 2–6 months, with at least 2 check-ups per year including a full sleep study with NIV. Overnight SpO_2_ with PtcCO_2_ recording during NIV is recommended at each visit as numerous asymptomatic patients remain hypercapnic during sleep with NIV despite a normal overnight SpO_2_ and normal daytime blood gases (56). This residual nocturnal hypercapnia can be easily corrected by simple measures such as changing the interface or the ventilator settings (56). Check-ups can also be performed at home with adequate training of staff (57). The simultaneous analysis of the in-built software of the ventilator and the overnight gas exchange gives useful information on important issues such as objective compliance, unintentional leaks, respiratory rate, airway pressure and residual respiratory events, which is particularly useful for centers which have a limited access to sleep studies (58, 59).

## What Are the Side Effects and Limitations of NIV?

NIV may be associated with side effects. The most common are those caused by the interface and comprise skin injury due to pressure sores, eye irritation due to unintentional air leaks, and facial deformity in young children (60). Skin injury is less common due to the improvements in interfaces for children and the use of “minimal-contact” interfaces such as nasal pillows. Mouth leaks may be minimized by the use of a pacifier in infants, or the change for a nasobuccal mask. Facial flattening and maxillary retrusion are the major side effects in young children and may add an obstructive component to the child's restrictive lung disease (60). These facial deformations may be prevented by the use of nasal pillows or minimized by alternating different interfaces such as a nasal and a nasobuccal mask. Other side effects are less common and may be caused by the positive pressure. Aerophagia, gastric distension, and oeso-gastric reflux may be observed with high inspiratory pressures or in case of patient-ventilator asynchrony. These abdominal side effects may be managed by decreasing the airway pressures, and/or correcting patient-ventilator asynchrony, and/or the use of an abdominal girdle. Unintentional leaks may be associated with discomfort, poor NIV tolerance and poor sleep quality with arousals (61).

In children requiring NIV during daytime, mouthpiece ventilation may be impossible in young children and difficult to accept in older children. Even if NIV is very effective, it may be difficult to apply “around the clock” on a long term basis. Some expert teams have a successful experience with NIV in totally ventilator-dependent children with NMD (33), but others consider that the child should be able to breathe spontaneously without NIV for a minimal time per day in order to be able to be safely maintained at home. A tracheostomy represents a possible option for children with a progressive NMD that has to be prepared and discussed thoroughly with the child and his parents. Within this context, determining the best interests of a child has been described as “balancing benefit and burdens of treatment and outcomes, whilst considering ascertainable wishes, beliefs and values and preferences of the child and their family, the cultural and religious views of the matter, the views of those providing care for the child and what choice is less restrictive for future options” (62). In some children and their families, the transition from NIV to a tracheostomy may be associated with a too high burden with regard to the improvement in quality of life of the child and his family (63). NIV may then be used as a part of a palliative care approach, without prolonging excessively a poor or unbearable quality of life. However, this remains a complex and evolving area of health-related quality of life (64–67). Of note, a tracheostomy was not always associated with a decrease in quality of life as evaluated by the patients themselves (68).

## Conclusion

Long term NIV is an extremely efficacious type of non-invasive respiratory support which has transformed the scope of chronic respiratory failure and severe sleep-disordered breathing in children with NMD by avoiding tracheotomies and allowing the child to live at home with a good quality of life for the child and his family. The tremendous heterogeneity of the disorders, ages, prognosis and outcomes of the patients underlines the necessity of a management by experienced, multidisciplinary pediatric centers, having technical competencies in pediatric NIV, and an expertise in sleep studies and therapeutic education.

## Author Contributions

BF was the main author of the manuscript. AA, SK, LG, AL, and TT contributed to the management of the children with neuromuscular disease followed in our center and the writing of the manuscript. All authors approved the final version of the manuscript.

## Conflict of Interest

The authors declare that the research was conducted in the absence of any commercial or financial relationships that could be construed as a potential conflict of interest.
